# Burden of Fluoroquinolone Resistance in Clinical Isolates of *Escherichia coli* at the Ho Teaching Hospital, Ghana

**DOI:** 10.4314/ejhs.v32i1.11

**Published:** 2022-01

**Authors:** John Gameli Deku, Kwabena Obeng Duedu, Emmanuel Ativi, Godsway Edem Kpene, Patrick Kwame Feglo

**Affiliations:** 1 Department of Medical Laboratory Sciences, School of Allied Health Sciences, University of Health and Allied Sciences, Ho, Ghana; 2 Department of Biomedical Science, School of Basic and Biomedical Sciences, University of Health and Allied Sciences, Ho, Ghana; 3 Department of Clinical Microbiology, School of Medicine and Dentistry, KNUST, Kumasi, Ghana

**Keywords:** Fluoroquinolone, resistant genes, Escherichia coli, ciprofloxacin, quinolone resistance(qnr) gene

## Abstract

**Background:**

The growing burden of antibiotic resistance is a threat to the management of infections. Infections by Escherichia coli are routinely treated with fluoroquinolone antimicrobial agents. Due to their frequent use, there has been increasing resistance to these drugs. We set out to determine the burden of fluoroquinolone resistance among clinical E. coli isolates at the Ho Teaching Hospital, Ghana.

**Methods:**

This was a cross-sectional study conducted from July 2018 to June 2019. One hundred and thirty-five E. coli isolates were cultured from various clinical samples. Antimicrobial susceptibility testing was performed using the Kirby-Bauer disk diffusion method with discs of nalidixic acid (NAL), ciprofloxacin (CIP), norfloxacin (NOR) and levofloxacin (LEV). Deoxyribonucleic acid (DNA) was extracted from the resistant isolates for the detection of fluoroquinolone resistant genes by polymerase chain reaction.

**Results:**

Ninety of the 135 isolates (66.7%) were resistant to at least one of the four fluoroquinolone drugs investigated. Resistance to NAL, CIP, NOR, and LEV was 51.0%, 51.1%, 38.8% and 35.7% respectively. Out of the fluoroquinolone resistant isolates, 69 carried one or more fluoroquinolone resistant genes. The predominant resistant genes were aac(6′)-Ib-cr (48.9%) and qnrD (25.6%). Seven of the isolates carried both qnrS and aac(6′)-Ib-cr genes. Two isolates carried 5 different fluoroquinolone resistant genes.

**Conclusion:**

High prevalence of resistance to 4 fluoroquinolone drugs was recorded with associated resistant genes. This is a threat to current efforts to control the spread of antibiotic resistance and calls for concerted efforts to curb the spread of these resistant organisms.

## Introduction

*Escherichia coli* is responsible for most of the Gram-negative infections clinically. Infections caused by this organism are routinely treated with fluoroquinolone antimicrobial agents. Additionally, fluoroquinolones are also used to treat Gram positive bacterial infections globally (1). These antimicrobials are broad-spectrum agents and are also used in veterinary practice. Due to their frequent use, there has been increasing resistance to these drugs (2).

Mechanisms of bacterial resistance to quinolones and fluoroquinolones are by both chromosomal and plasmid mediated (3). Three resistance mechanisms have been documented: (i) mutational alterations in the target enzymes (DNA gyrase and topoisomerase IV) (4), (ii) modification of fluoroquinolones with piperazinyl substituents by Aac(6′)-Ib-cr aminoglycoside transferase (1) and (iii) increase in the minimum inhibition concentrations (MICs) of ciprofloxacin by efflux pump proteins (3). These efflux pumps pump out the fluoroquinolone drug molecules from the bacteria, leading to the reduction in the concentration of the drug in the bacterial cells. The bacteria then become less susceptible to the fluoroquinolone compound, resulting in an increase in the MIC. Some studies have reported an increase in the MICs of nalidixic acids by four to eight folds (5, 6) and ciprofloxacin by eight to thirty-two folds (7) due to the genetic mechanisms of resistance.

In Ghana, there is paucity of information on fluoroquinolone resistance and resistance-determining genes. This study therefore investigated both phenotypic and associated molecular mechanisms of resistance of *E. coli* to selected fluoroquinolone agents in Ho Teaching Hospital, Ghana.

## Methods

**Collection of *Escherichia coli* isolates**: This was a cross-sectional study involving 135 non-repetitive *Escherichia coli* strains that were isolated from various clinical specimens produced by 135 patients who visited Ho Teaching Hospital from July 2018 to June 2019. The specimens were cultured on MacConkey agar and blood agar. These specimens included urine, blood, ear swab, high vaginal swabs, among others. Isolation and identification of the isolates were based on colonial characteristics, Gram stain reaction, triple sugar fermentation test, indole test, citrate utilization test, urea utilization test, Voges Proskauer and methyl red tests (6). The isolated *E. coli* isolates were preserved in 80% glycerol-Mueller Hinton broth and stored in a -80 freezer. The stored isolates were revived and used for the determination of the resistotypes and resistant genes. *Escherichia coli* ATCC 25922 and *Klebsiella pneumoniae* NCTC 13442 were used as control organisms.

**Antimicrobial susceptibility testing on the *E. coli* isolates**: Antimicrobial susceptibility testing was done on all the 135 *E. coli* isolates using the Kirby-Bauer disk diffusion method as specified by Clinical and Laboratory Standards Institute (CLSI) guidelines (7). In the performance of this test, three discrete colonies of the test organism were touched with a sterile straight wire and emulsified in buffered phosphate saline and the turbidity measured with a Densi CHEK plus densitometer (Biomerieux, U.S.) to obtain a turbidity of 0.5 McFarland. Using a sterile swab, Mueller-Hinton agar (Oxoid, UK) was seeded with the test organism. Antibiotic disks were placed on the surface of the seeded agar using a pair of sterile forceps. The organisms were tested against ciprofloxacin (5µg), Levofloxacin (5µg), Nalidixic acid (30µg) and Norfloxacin (10µg). After incubating aerobically at 37°C for 16–18 hours, the diameter of inhibition zones was measured using a ruler. The inhibition zones were then compared with a set of standard interpretative chart according to CSLI (7), to determine if the *E. coli* isolates were susceptible, intermediately susceptible or resistant. *Escherichia coli* ATCC 25922 was used as quality control organism.

**Detection of Fluoroquinolone Resistant Genes by Polymerase Chain Reaction (PCR)**

**Revival of isolates and DNA Extraction**: The isolates were retrieved from the freezer by scraping the surface onto nutrient agar. The plate was streaked and incubated overnight. A colony was picked and inoculated into 30ml Luria Bertani broth (Oxoid, UK) and incubated overnight. Genomic DNA was extracted from the overnight culture using high molecular weight phenol-chloroform extraction method (8) except that Tris EDTA (TE) buffer was used as the elution buffer. The concentration of the extracted DNA was measured using NanodropOne Spectrophotometer (Thermo Scientific).

**Detection of fluoroquinolone resistant genes by singleplex PCR**: Fluoroquinolone resistant genotypes were determined by PCR using the Quickload OneTaq 2x Master Mix (NEB, UK). Oligonucleotide primers were used to detect the presence of gene loci for *aac(6′)-Ib-cr, qepA*, *qnrA, qnrB, qnrC, qnrD* and *qnrS*. The PCR conditions used were: 94°C for 30 seconds followed by 35 cycles of reaction at 94°C for 30 seconds, 55°C (for *aac(6′)-Ib-cr*) for 45 seconds and 68°C for 60 seconds/kb of product and a final extension at 68°C for 5 minutes. Annealing temperatures for the other products were 51 °C for *qepA*, 53°C for *qnrA, qnrB* and *qnrS*, 52 °C for *qnrC* and 50 °C for *qnrD*. The primer sequences and the sizes are listed in table 1. A total of 12.5µl reaction volume was used which comprised of 6.25µl of OneTaq quick load 2x master mix with standard buffer (NEB, USA), 0.25µl each of forward and reverse primers, 1µl of DNA template and nuclease-free water (4.75µl). In-house generated control DNA from *E. coli* were used as quality control materials. The PCR products were loaded onto agarose gel prestained with GelRed nucleic acid gel stain (Sigma, GmbH), electrophoresed and visualized using a UVITEC (Cambridge, UK) gel imager.

**Table 1:** Primers used to detect fluoroquinolone resistant genes in *E. coli*

Target gene	Primer	Sequence (5′-3′)	Amplification product (bp)	Reference
*qnrA*	qnrA_F	ATTTCTCACGCCAGGATTTG	516	(37)
	qnrA_R	GATCGGCAAAGGTTAGGTCA		
*qnrB*	qnrB_F	GGMATHGAAATTCGCCACTG	246	(38)
	qnrB_R	TTTGCYGYYCGCCAGTCGAA		
*qnrC*	qnrC_F	GGGTTGTACATTTATTGAATC	447	(38)
	qnrC_R	TCCACTTTACGAGGTTCT		
*qnrD*	qnrD_F	CGAGATCAATTTACGGGGAATA	582	(38)
	qnrD_R	AACAAGCTGAAGCGCCTG		
*qnrS*	qnrS_F	ACGACATTCGTCAACTGCAA	417	(37)
	qnrS_R	TAAATTGGCACCCTGTAGGC		
*qepA*	qepA_F	CTGCAGGTACTGCGTCATG	403	(38)
	qepA_R	CGTGTTGCTGGAGTTCTTC		
*aac(6′)-* *Ib-cr*	aac (6′)-Ib-cr_F	TTGCGATGCTCTATGAGTGGCTA	482	(38)
	aac(6′)-Ib-cr_R	CTCGAATGCCTGGCGTGTTT		

**Ethical considerations**: Permission to carry out this study was granted by the management of Ho Teaching Hospital with Ref No. VRH/1/102. Ethical clearance for the study was granted by the Joint Committee on Human Research, Publication and Ethics, School of Medicine and Dentistry, Kwame Nkrumah University of Science and Technology and the Komfo Anokye Teaching Hospital, Kumasi, with the protocol number CHRPE/AP/204/18.

**Statistical analysis**: All statistical analyses were performed using IBM-SPSS Statistics for Windows, version 26 (IBM Corp., Armonk, N.Y., USA) and GraphPad Prism 8.0 (GraphPad software, Inc., San Diego, CA). Descriptive outcomes for categorical variable were presented in summary tables as frequencies and percentages. Chi-square and Fisher's Exact tests were done for categorical variables for differences in proportions with P-values of <0.05 considered statistically significant.

## Results

This study analyzed 135 *E. coli* isolated from clinical specimens at the Ho Teaching Hospital. Majority of the isolates (82.2%) were obtained from female participants and were mostly obtained from the 30–39 years age group 32(23.7%). Most of these isolates being 130(96.3%) were recovered from residents of the Ho township. Majority of these are E. *coli* isolates from urine specimens as presented in Table 2.

**Table 2:** Socio-demographic characteristics of patients

Parameter	Number of isolates	Percent
**Gender**		
Male	24	17.8
Female	111	82.2
**Age**		
< 10 years	11	8.2
10–19 years	2	1.5
20–29 years	26	19.3
30–39 years	32	23.7
40–49 years	19	14.0
50–59 years	16	11.8
≥60 years	29	21.5
**Marital Status**		
Single	46	34.1
Married	84	62.2
Widowed	2	1.5
Co-habiting	3	2.2
**Religion**		
Christian	128	94.8
Muslim	2	1.5
None	5	3.7
**Employment**		
None	53	39.3
Formal	34	25.1
Informal	48	35.6
**Location**		
Ho	130	96.3
Outside Ho	5	3.7
**Specimen type**		
Urine	98	72.6
Wound swab	14	10.4
High Vaginal Swab	10	7.4
Blood	5	3.7
Ear swab	5	3.7
Sputum	2	1.5
Pleural aspirate	1	0.7

**Figure 1:**
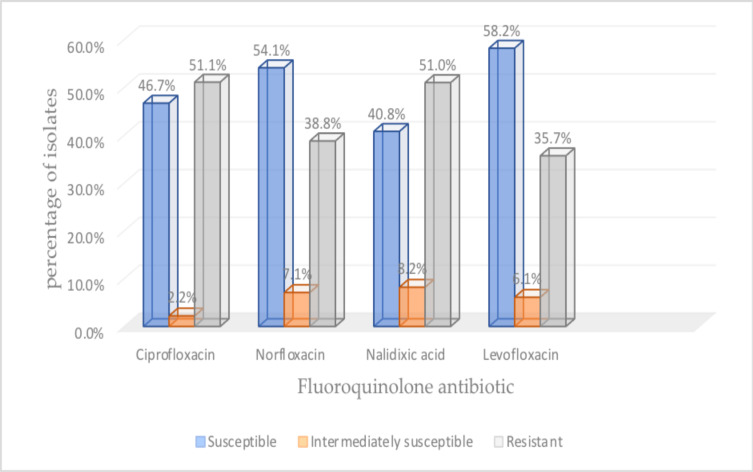
Antibiotic susceptibility and resistant patterns of selected fluoroquinolone antibiotics.

There were 51.0% of the isolates resistant to nalidixic acid, 51.1% to ciprofloxacin, 38.8% to norfloxacin and 35.7 to levofloxacin. Overall, 66.7% were resistant to at least one of the four drugs tested. Details of these results are presented in Figure 1.

Sixty-nine (76.7%) of the phenotypically fluoroquinolone resistant isolates carried at least one fluoroquinolone resistant determinant gene. The presence of fluoroquinolone resistant genes was higher among isolates from the male participants (80.0%) than was seen for isolates from their female counterparts (75.7%). More men were infected with ciprofloxacin resistant *E. coli* than females and the difference was significant (p=0.0127). All the participants between the ages of 10 and 19 were infected with nalidixic acid resistant E. coli. More men (80.0%) were infected with isolates carrying fluoroquinolone resistant genes as compared to their female counterparts. Again, majority (83.3%) of the E. coli carrying resistant genes were isolated from participants below 10 years and those between 40 and 49 years as presented in Table 3.

**Table 3:** Phenotypic and genotypic distribution of quinolone resistance stratified by socio-demographic and patients characteristics

Socio- demography/ patients' characteristics	Total	CIP (n=135)		NAL (n=98)		LEV (n=98)		NOR (n=98)			Quinolone resistant gene		

		Resistant	P- value	Resistant	P- value	Resistant	P- value	Resistant	P- value	Total	Present	Absent	P- value
**Total**	**135**	**69**		**50**		**35**		**38**		**90**	69(76.7)	21(23.3)	
**Gender**													
Male	**24**	18(75.0)	**0.0127**	5(50.0)	0.1016	5(45.5)	0.6158	5(50.0)	0.4599	**20**	16(80.0)	4(20.0)	0.7737
Female	**111**	51(46.0)		45(51.7)		30(34.5)		33(39.3)		**70**	53(75.7)	17(24.3)	
**AGE**													
< 10 years	**11**	5(45.5)	0.4376	2(28.6)	0.3438	3(42.9)	0.8141	2(28.6)	0.8899	**6**	5(83.3)	1(16.7)	0.9198
10–19 years	**2**	0(0.0)		2(100.0)		0(0.0)		0(0.0)		**2**	1(50.0)	1(50.0)	
20–29 years	**26**	13(50.0)		11(57.9)		8(42.1)		9(47.4)		**17**	12(70.6)	5(29.4)	
30–39 years	**32**	16(50.0)		9(40.9)		7(31.8)		9(40.9)		**20**	16(80.0)	4(20.0)	
40–49 years	**19**	10(52.6)		7(46.7)		7(46.7)		5(33.3)		**12**	10(83.3)	2(16.7)	
50–59 years	**16**	6(37.5)		7(53.9)		3(21.4)		4(33.3)		**10**	7(70.0)	3(30.0)	
>60 years	29	19(65.5)		12(63.2)		7(36.8)		9(52.9)		**23**	18(78.3)	5(21.7)	
**Location**													
Ho	130	68(52.3)	0.2016	49(52.1)	0.651	35(36.8)	0.3266	38(41.8)	0.3213	**90**	69(76.7)	21(23.3)	1.000
Outside Ho	5	1(20.0)		1(33.3)		0(0.00)		0(0.00)		**0**	0(0.0)	0(0.00)	

CIP - Ciprofloxacin, NAL – Nalidixic acid, NOR – Norfloxacin, LEV – Levofloxacin P-value is significant at <0.05.

**Figure 2:**
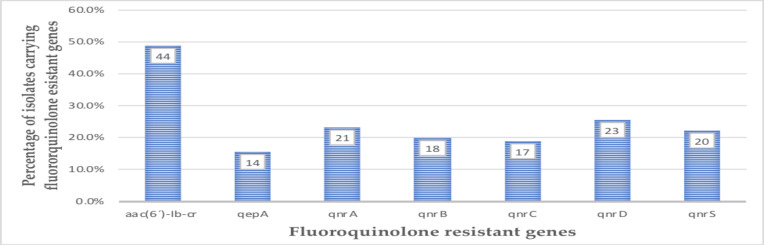
Prevalence of Fluoroquinolones resistant genes

The *aac(6′)-Ib-cr* was the most frequently identified resistant gene in 44 of the isolates, representing 48.9% of the isolates, with the least carriage being *qepA* 14(15.6%), as shown in Figure 2. Furthermore, the *aac(6′)-Ib-cr* was the predominant gene identified in isolates resistant to Norfloxacin (57.9%) and Levofloxacin (57.1%). The *qnrC* was also detected in only 4(10.5%) Norfloxacin-resistant *E. coli* isolates. There was no significant association between fluoroquinolone resistance and resistant genotype. Details of these results are presented in Table 4.

**Table 4:** . Distribution of fluoroquinolone resistant genes among *E. coli* isolates.

Fluoro- quinolone antibiotic	Number of resistant isolates	qnr A	P- value	qnr B	P-value	qnr C	P-value	qnr D	P- value	qnr S	P- value	qep A	P- value	aac (6′)- Ib-cr	P- value
CIP	69	16(23.2)	0.4329	16(23.2)	0.5748	12(17.4)	0.7465	19(27.5)	0.9987	14(20.3)	0.7082	13(18.8)	0.9437	35(50.7)	0.8639
NOR	38	5(13.2)		5(13.2)		4(10.5)		11(29.0)		11(29.0)		6(15.8)		22(57.9)	
NAL	50	7(14.0)		8(16.0)		7(14.0)		14(28.0)		12(24.0)		8(16.0)		26(52.0)	
LEV	35	5(14.3)		6(17.1)		4(11.4)		10(28.6)		10(28.6)		7(20.0)		20(57.1)	

CIP - Ciprofloxacin, NAL – Nalidixic acid, NOR – Norfloxacin, LEV – Levofloxacin.

The study showed that 19 of the isolates had one resistant gene, but multiple fluoroquinolone resistant genes were detected in 25 of the isolates which had at least 2 different resistant genes. Five different resistant genes were also detected in 2 of the isolates.

## Discussion

Fluoroquinolone resistance in *Escherichia coli* is evolving as a crisis. Infections caused by *E. coli* are generally treated with fluoroquinolones because of their broad-spectrum activity and efficacy (9). However, the usefulness of this class of antibiotics in Ghana is in doubt due to the increasing resistance being recorded in certain studies. A study conducted by Asafo-Adjei et al (10) among bladder outlet obstruction patients in Accra, Ghana recorded a high resistance to Nalidixic acid (82.8%) and Ciprofloxacin (75.0). In the current study, the resistant proportions of isolates were slightly lower for Nalidixic acid, Levofloxacin, Norfloxacin and Ciprofloxacin in isolates recovered from both urine and non-urine specimens. Similar results were recorded in other study centers across the country (11–13). The high prevalence of resistance to fluoroquinolones recorded in this study could be attributed to their high usage and misuse in Ghana.

The rate of Ciprofloxacin resistance from this study is similar to what was reported in a countrywide antibiotic resistance survey in Ghana in 2015 where more than 50% of the isolates were resistant to this antibiotic (14). Ciprofloxacin is mostly prescribed fluoroquinolones in Ghana for empiric treatment of bacterial infections (15). This is due to the high level of susceptibility of bacterial pathogens, such as *E. coli* to this antibiotic in the past (10). Furthermore, in many formal and informal health facilities in many African countries, Ciprofloxacin has been the drug of choice in treating bacterial infections (16). The high level of resistance to this antibiotic in this study calls for a review of its usage in the empiric treatment in Ghana, since fluoroquinolones treatment are no longer effective in majority of the patients in several countries across the globe (17).

Plasmids play a vital role in the resistance to quinolones and fluoroquinolones because they carry transferable resistant genes from one bacterium to the another and can be within or between species. These resistant genes are common among *Enterobacteriaceae*, especially *E. coli* and *Klebsiella spp.* (18). The genes for fluoroquinolone resistance are known to be both chromosomal and plasmid-mediated making the high prevalence situation very worrying. The *aac(6′)-Ib-cr* gene which occurred commonly in our isolates have also been reported to have a link to extended spectrum beta-lactamases action against non-beta-lactam antibiotics (19–21). The 63.8% prevalence of *aac(6′)-Ib-cr* in this study was lower than that found in other studies from Uruguay (64.3%) (22), Egypt (66.7%) (23) and Ghana (100.0%) (24). On the contrary, lower prevalence in comparison to our current finding was reported from other studies (25, 26). Even though *aac(6′)-Ib-cr* gene is noted for producing low-level resistance by itself, it plays an important role in acquisition of a clinical level of resistance to ciprofloxacin and norfloxacin, when combined with three or four chromosomal mutations, both in vitro and in vivo (27).

The *qepA* efflux pump gene recorded in this study has also been reported as plasmid-mediated quinolone resistance (PMQR) mechanism (28, 29). This prevalence of the gene in this study was lower than that reported by Haggaget al (17) in Egypt and in Ghana (24).

Another PMQR gene was the quinolone resistance (*qnr)* gene. The Qnr encoding proteins block the action of fluoroquinolone antibiotics on DNA gyrase and topoisomerase IV (9). The 3 main Qnr encoding proteins are QnrA, QnrB, and QnrS (30, 31). In a study by Mensah-Attipoe et al (24) aimed to molecularly characterize Ciprofloxacin-resistant *E. coli*, all the 3 main qnr encoding proteins were detected. Although *qnrD* was not detected in the earlier studies, it was found in the current study. This is very worrying, as all the fluoroquinolone drugs may be out of use in the very near future due to their non-efficacy in treating bacterial infections.

The *qnr* genes recorded in this study were lower than the 55.1% (for *qnrA* and *qnrB*), 75.8% (for *qnrC*) and 89.6% (for *qnrS*) reported by Mensah-Attipoe et al (24) in Ghana in their study to characterize ciprofloxacin resistant *E. coli*. In that study, the researchers used 440 stored *E. coli* isolates collected from 24 different laboratories dotted all over the country. The multi-center approach used by these researchers together with the comparative higher sample size might have contributed to the differences in the prevalence of qnr genes recorded in this study. The difference in the prevalence could also be due to geographical locations of the two study sites.

Another mechanism is chromosomal mutations of *gyrA* or *parC* genes or due to impermeability of the membranes (32). *E. coli* resists the action of fluoroquinolone by development of chromosomal mutations mainly in the quinolone-resistance determining regions of the target genes; *gyrA* which encodes DNA gyrase and *parC* which encodes topoisomerase IV (32). Mutations at serine-83 (Ser-83) and asparagine-87 (Asp-87) in the *gyrA* gene are among the most often observed mutations in *E. coli* mutant strains (33). Resistance due to mutations in *gyrB* and *parE* are less frequent than those in *gyrA* and *parC* (34, 35). Mutations that result in a reduction in the intracellular concentration of the antibiotic either by decreased uptake due to porin loss or reduced membrane permeability, or increased efflux activities, or a combination of both, can confer quinolone resistance (1, 36).

*E. coli* isolates resistant to the fluoroquinolones tested carried the seven resistant genes tested. This mean that there may be multiple resistant mechanisms against one particular antimicrobial agent, making treatment of such infections very difficult. In this study, some isolates carried more than one different fluoroquinolone resistant genes. Alternatives treatment with other antimicrobial agents in the same class of antibiotics are more likely to be of little value due to the multiple gene carriage.

In conclusion, high proportion of *E. coli* isolated from Ho Teaching Hospital are resistant to fluoroquinolones. Resistant genes determining resistance to the fluoroquinolones were detected in the isolates with *aac(6′)-Ib-cr* being the most commonly detected genes. The heavy use of this class of antimicrobial agents may obliterate their importance.

The study is limited in that only four quinolone and fluoroquinolone antibiotics routinely used in Ghana were included in the study. Again, the study was a single-center study and does not reflect the situation in the entire country although it suggests what could be expected fluoroquinolone resistance in Ghana. It is therefore recommended that this study is repeated in other parts of the country to establish a national fluoroquinolone prevalence with addition of other fluoroquinolone antibiotics.
